# Accuracy in CPAA for C, N and O and in ERDA and NRA for H

**DOI:** 10.6028/jres.093.126

**Published:** 1988-06-01

**Authors:** T. Nozaki

**Affiliations:** Department of Hygienic Sciences, Kitasato University, Kitasato, Sagamihara, Kanagawa 228, Japan

## 1. Introduction

Trace amounts of H, C, N and O can be determined by only a few methods, often with rather poor accuracies. Charged particle activation analysis (CPAA) is highly reliable for C, N and O, and is used for calibration of other methods. Elastic recoil detection analysis (ERDA) and nuclear reaction analysis (NRA) have recently been utilized for H and D near the surface. Accuracies in these analyses are discussed on the basis of our experimental data.

## 2. Accuracy in CPAA

### 2.1 Advantage and Error in CPAA

The advantage of CPAA for C, N and O consists of inherent high sensitivity, freedom from various contaminations, and reliability in the use of comparators. The following reactions are used for activation: ^12^C(^3^He,α)^11^C or ^12^C(d,n)^13^N; ^14^N(p,α)^11^C; and ^16^O(^3^He,p)^18^F. Uncertainty in the measurement of bombarding particle beam current frequently becomes a major cause of error in CPAA. Also, some bombardment damage of the sample often results in noticeable overestimation of C and O.

### 2.2 Precision for O and C

We analyzed O in a silicon wafer repeatedly nine times from 1982 to 1985 by the nondestructive measurement of the ^18^F annihilation radiation with a Ge(Li) detector [1]. The results gave the mean value of 10.04 ppm wt with *σ* = 0.18 ppm.

We examined chemical separation of ^11^C in Si, using ^11^C-containing Si prepared by proton bombardment of B-doped Si [1]. The following separation method was selected: alkali-dissolution of the pulverized sample; KMnO_4_ oxidation of the ^11^C under microwave heating to 700 °C; and generation of ^11^CO_2_ and its conversion into Li_2_^11^CO_3_. The measurement of ^11^C activity before and after the separation and of carrier recovery indicated that 9.0±2.0% of the ^11^C apparently disappeared. Our results obtained by this separation has thus been corrected for this 9%.

For the separation of ^13^N, dry fusion into ^13^N_2_ and wet distillation as ^13^NH_3_ are used [1]. Carbon in 27 plates of GaAs made from a single rod were analyzed by the dry method in different machine times of two cyclotrons. The mean results were 24.6 ppb wt with *σ* = 2.6 ppb. For the last five plates taken from adjacent parts of the rod, the results were 23.254±0.84 ppb. We separate ^18^F by precipitation as KB^18^F_4_ [2]. This method has shown satisfactory precision.

### 2.3 Calibration of IR Spectrophotometry

**Figure 1:**
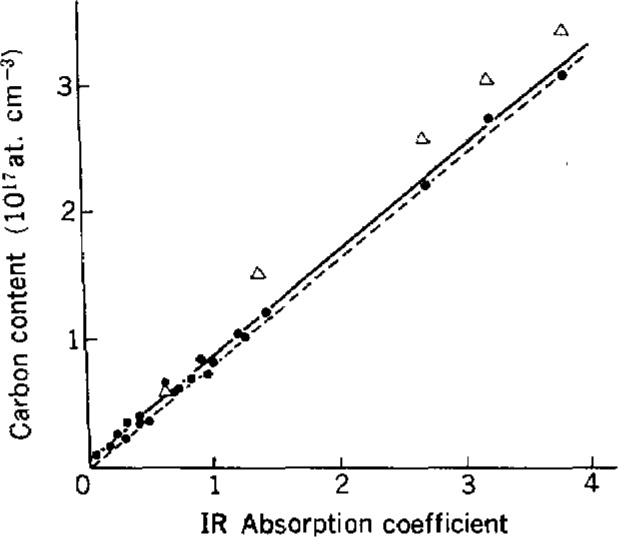
Calibration curve for IR spectrophotometry of C in Si (605 cm^−1^, at room temperature). Solid line, before correction for C in IR reference Si; (C concentration, 4.3 × 10^15^ at cm^−3^); broken line, after the correction; Δ, results of SIMS.

Our calibration curve for IR spectrophotometry of C in Si is shown in figure 1 [1]. About 70 carefully prepared samples were submitted to round-robin IR measurement in 24 organizations. About 1/3 of them were then analyzed by CPAA; some of them were also analyzed by SIMS. The differences between the results of CPAA and SIMS are probably caused by the presence of background in SIMS. Satisfactory calibration curves were obtained also for O and N in Si [3,4]. Efforts are now being made to obtain a reliable calibration curve for C in GaAs.

## 3. Accuracy for ERDA and NRA for H

**Figure 2:**
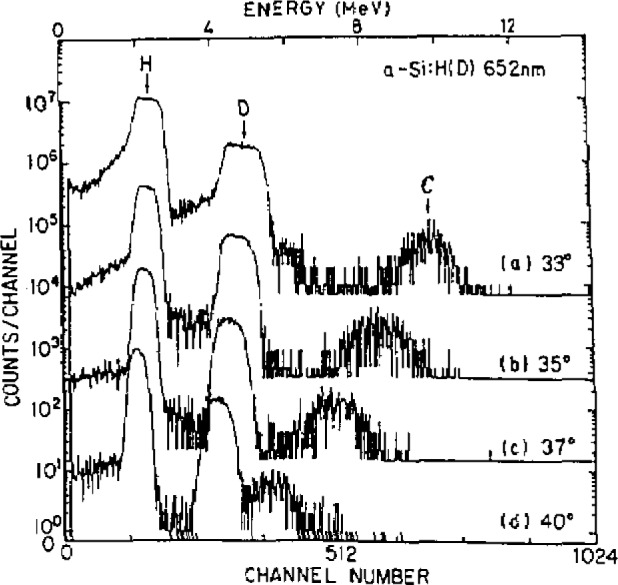
ERDA spectra for various detector angles. Sample: H- and D-containing amorphous silicon film 652 nm thick; incident particle: 42.3 MeV Ar; target angle: 30 °C; absorber: 10 μm Al foil.

Figure 2 shows ERDA spectra of a H- and D-containing amorphous silicon film on a Si wafer [5]. The detection efficiency is seen to depend highly on the detection angle. No suitable substances are found as comparators for H and D, and their quantities are usually obtained in comparison with an internal standard element by the use of the theoretical relationships and experimental parameters. The H content of amorphous silicon films obtained by ERDA and vacuum fusions are given in table 1 [5]. At present, the accuracy for H by ERDA is notably lower than for C, N and O by CPAA. The depth profiling of H and D is possible by ERDA, as is seen in figure 2; but further studies are necessary.

In the depth profiling of H by the ^1^H+^15^N→^12^C+*γ*(4.44 MeV) reaction, it is easy to get the shape of the profile reproducibly but difficult to obtain an absolute value for H within a 20% uncertainty. We now use D as an activatable tracer, determining it by the reactions D+^3^He→α+p(14 MeV) and D+^15^N→^16^O+n(or p+*β*^−^)+*γ*(6.13, 7.11 MeV) [6]. Experimental data are required concerning the accuracy of these methods.

## 4. Conclusion

At present, accuracy can be determined for the CPAA of C, N and O. For the ERDA and NRA determination of H and D, experimental data are still too scarce for consideration of accuracy.

## Figures and Tables

**Table 1:** Hydrogen content of amorphous silicon films measured by three methods (1017 atoms/cm^2^): ERDA, vacuum extraction with the measurement of volume (VOL) and heat conductivity (HC)

Film thickness (nm)	ERDA	VOL^a^	HC^b^	VOL/ERDA	HC/ERDA
1065	7.36	7.92	7.38	1.08	1.00
830	5.77	6.54	5.90	1.17	1.06
672	4.25	5.60	5.11	1.32	1.20
650	3.78	5.26	4.40	1.39	1.16
526	4.53	4.40		0.97	
510	3.96	3.63		0.92	
310	2.40	3.07	2.67	1.28	1.11
270	1.85	2.19	1.89	1.18	1.02

Average				1.16	1.09
				± 0.17	± 0.08

a By Prof. Ogawara, Science Univ. of Tokyo.

b By Dr. Nomura, Mitsubishi Metals Co.
